# Knowledge, attitude and perception of Pakistanis towards COVID-19; a large cross-sectional survey

**DOI:** 10.1186/s12889-020-10083-y

**Published:** 2021-01-05

**Authors:** Zoya Fatima Rizwan Ladiwala, Rubaid Azhar Dhillon, Ibrahim Zahid, Omar Irfan, Muhammad Sharjeel Khan, Safia Awan, Javaid Ahmad Khan

**Affiliations:** 1 Dr. Ruth K. M. Pfau Civil Hospital Karachi, Karachi, Pakistan; 2 Islamic International Medical College, Rawalpindi, Pakistan; 3 McMaster University, Hamilton, Canada; 4 Hospital for Sick Children, Toronto, Canada; 5 Alumni, Aga Khan University, Stadium Road, Karachi, Pakistan; 6 Altamash Institute of Dental Medicine, Karachi, Pakistan; 7 Department of Medicine, Aga Khan University Hospital, Karachi, Pakistan; 8 Pulmonary and Critical Care Medicine, Aga Khan University, Karachi, Pakistan

**Keywords:** Coronavirus, Pandemic, Pakistan, Attitude, Perception, Knowledge

## Abstract

**Background:**

The Novel Coronavirus Disease (COVID-19) has created havoc globally as countries worldwide struggle to combat this pandemic. Since prevention and social isolation are known to be the only ways to prevent the spread of COVID-19, this has created challenges among the lower-middle income countries (LMIC) including Pakistan, as it battles between an under-resourced healthcare, an economic shutdown, and widespread myths and misconceptions. Therefore, a study was conducted to evaluate the knowledge, attitude and perceptions regarding COVID-19 as public understanding is vital to help facilitate the control of this outbreak.

**Methods:**

A pre-validated online questionnaire was distributed among the general population of Pakistan from 1st to 12th June 2020. Descriptive statistics were analyzed using SPSS v25. Adequate knowledge was assigned as a score of > 4 (range: 0–8) and good perception as a score of > 3 (range: 0–5). Chi-square test was used to determine the significance of difference in knowledge and perception of COVID-19 with socio-demographic characteristics. Logistic regression analysis was run to identify factors associated with adequate knowledge and perception. *P* < 0.05 was considered as significant.

**Results:**

A total of 1200 respondents participated in this study with a wide range of age groups and education. Majority of the respondents had adequate knowledge (93.3%) with a mean score of 6.59 ± 1.35, and good perception (85.6%) with a mean score of 4.29 ± 0.82. Significant differences in knowledge and perception were observed among genders, age groups, education and between students and employees in the healthcare and non-healthcare department. A multivariate analysis revealed a higher educational status and female gender to be significant predictors of adequate knowledge and perception.

**Conclusions:**

Albeit the surge of COVID-19 cases in Pakistan, the participants demonstrated an overall adequate knowledge and good perception towards COVID-19. There is a need to follow the preventive protocols and dissemination of correct information through conducting educational interventions that target safe health practices and provide appropriate information on this infection.

**Supplementary Information:**

The online version contains supplementary material available at 10.1186/s12889-020-10083-y.

## Background

The rapidly evolving outbreak of the Novel Coronavirus Disease (COVID-19) caused by severe acute respiratory syndrome coronavirus 2 (SARS-CoV-2) has proven to be a matter of utmost concern amongst global authorities, as countries worldwide struggle to combat this pandemic. COVID-19 is an emerging respiratory illness that was first detected in Wuhan, China on 12^th^December 2019 [1]. As the world is thoroughly well-connected via trade, business and travel, the virus has since then managed to affect 212 countries and territories worldwide. This eventually led to the World Health Organization (WHO) declaring COVID-19 a public health emergency of international concern on 30th January 2020 and subsequently a global pandemic on 11th March 2020 [2].

The two concerning features of this virus are low pathogenicity and high transmissibility, which has led to an exceedingly high prevalence and fatalities caused by it, as compared to its predecessors [3]. The WHO report revealed a global mortality rate of COVID-19 to be 3.4% [4]. Currently, no specific treatment of COVID-19 exists and prevention and social isolation have been the only recognized ways to control the spread. Some evidence has emerged regarding the efficacy of Dexamethasone in critical cases of COVID-19, as reported from a recovery trial in the United Kingdom [5]. Nonetheless, high prevalence and no definite treatment has indeed created unimaginable challenges in the lower middle income countries (LMIC) where extended periods of lockdowns along with the lack of infrastructure, under- resourced healthcare and weak financial governance has led to crippling economies, escalating unemployment rates and an ever increasing burden on healthcare.

Pakistan, with a limited allotted health care budget lacks sufficient medical equipment such as ventilators, hospital gowns and personal protective equipment to combat the ongoing pandemic [6]. Despite strict measures enforced by the government, up until July 17, 2020, a grim total of 260,000 cases with over 5000 deaths have been reported nationally. The provincial governments enforced a nationwide lockdown during early March which was unfortunately partly opposed by hard-line clerics and religious activists who insisted citizens to continue routine congregational prayers at mosques [7]. During the course of this pandemic, several incidents of mass gatherings have been observed nationally, demonstrating poor knowledge of the outbreak.

The fragmented healthcare system of Pakistan coupled with the uncooperative attitude of the local population and the religious protestors to social distancing measures alongside the overall poor health literacy calls for an urgent strategic plan. Therefore, a study was conducted to assess the knowledge, attitude and perception of the Pakistani population towards COVID-19, and to identify factors affecting the knowledge and perception scores among the residents. Through this study, authors aimed to help raise awareness and clear misconceptions regarding COVID-19, preventing the already -stretched healthcare system of Pakistan from being overwhelmed.

## Methods

An adaptive cross-sectional study was conducted among the general population of Pakistan from 1st to 12th June 2020 to assess their knowledge, attitude and perceptions regarding COVID-19.The Ethical Review Committee of Islamic International Medical College, Rawalpindi, Pakistan approved the study protocol before the initiation of the formal survey.

Due to the mode of transmission through human interaction of SARS-CoV-2, an online medium to collect responses was adopted. The questionnaire was originally produced in the English language but was also translated to Urdu by the research investigators in order to interview participants orally who were non-fluent in English. This was then back-translated to ensure validity. The targeted population, selected on the basis of non-probability convenience sampling, consisted of participants aged 15 years and above who were permanent residents of Pakistan and wished to voluntarily take part in the study. A written parental consent was obtained on behalf of participants below the age of 16. The use of implied consent to participate upon completion of the questionnaire was approved by the Ethical Review Committee. The aim of the study along with the consent form was attached with the questionnaire; those who were not willing to participate were excluded.

A self-administered questionnaire, designed after going through previously validated questionnaires from similar published studies [8, 9] and online published surveys was formulated as a tool for data collection. A pilot study among ten health care professionals was conducted to ensure clarity, relevance and compliance, following which necessary changes were made accordingly. These responses were excluded from the final results. The survey comprised of 33 questions which was divided into three sections: 1) Socio-demographic characteristics, 2) Knowledge and perceptions of COVID-19 and 3) Attitude and Practices towards COVID-19. Participants were also provided with an opportunity to view the correct answers at the end of the survey through which the goal of educating and raising awareness of the disease was accomplished.

The sample size was estimated using the software Epi-Info for the survey with 50% expected to lack knowledge on the subject. With an error bound of 0.03 (3%) and 95% confidence interval (CI), the maximum sample size was calculated as 683. This was further increased to a total of 1200 participants to ensure maximal representation of the population. Descriptive analysis was performed to calculate frequencies and percentages for the socio-demographic characteristics, knowledge, attitude, practices and perceptions towards COVID-19 using Statistical Package for the Social Sciences (SPSS) version 25. Results were reported as mean ± standard deviation for quantitative variables and frequencies (proportions) for qualitative variables. The responses to questions regarding knowledge and perception were scored as ‘1’ (correct) and ‘0’ (wrong), with scores ranging from 0 to 8 and 0 to 5 respectively. A cutoff level of ≤4 was considered as poor knowledge whereas > 4 as adequate knowledge. Likewise, perception was classified as good (score > 3) or poor (score ≤ 3). The comparison of adequate knowledge and perception among groups within the socio-demographics was assessed by using the Chi-square test or Fisher Exact test whereever appropriate. Univariate and multivariate analyses was performed to compare the knowledge and perception scores with each variable of interest. All *p*-values were two sided and considered as statistically significant if < 0.05. Cronbach’s alpha was used to assess internal consistency for each knowledge and perception scale.

## Results

A total of 1200 participants completed the online questionnaire out of total 1450 questionnaires which were distributed with a response rate of 82.7%. As shown in Table 1, most of the population was aged between 20 and 29 years (44.6%, *n* = 535). Majority of the respondents were unmarried (56.9%, *n* = 683) and were residents of Sindh province (77.8%, *n* = 934). More than half (58.5%, *n* = 702) of the sample had completed a bachelor’s degree. On the other hand, 38.1% (*n* = 457) of the participants were students, out of which the majority (57.5%, *n* = 263) were enrolled in a health care related field. Roughly, one quarter (22.7%, *n* = 273) of the population were either smokers or had quit smoking. A greater part of the sample (86.3%, *n* = 1036) had not contracted the COVID-19 virus.

**Table 1:** Demographic characteristics and comparison of adequate knowledge and perceptions *(n = 1200)*

	n (%)	Adequate knowledge; n (%)	*p* value	Good perception; n (%)	*p* value
Age					
15–19	123 (10.3)	113 (91.9)	0.001*	103 (83.7)	0.001*
20–29	535 (44.6)	517 (96.6)		483 (90.3)	
30–39	202 (16.8)	182 (90.1)		165 (81.7)	
40–49	136 (11.3)	127 (93.4)		114 (83.8)	
50–59	116 (9.7)	103 (88.8)		94 (81.0)	
60+	88 (7.3)	78 (88.6)		68 (77.3)	
Gender					
Male	459 (38.3)	404 (88.0)	< 0.001*	366 (79.7)	< 0.001*
Female	741 (61.8)	716 (96.6)		661 (89.2)	
Marital status					
Married	517 (43.1)	468 (41.8)	0.001*	420 (81.2)	< 0.001*
Single	683 (56.9)	652 (58.2)		607 (88.9)	
Education					
No education	26 (2.2)	19 (73.1)	< 0.001*	12 (46.2)	< 0.001*
Primary school	30 (2.5)	19 (63.3)		14 (46.7)	
Secondary school	201 (16.8)	181 (90.0)		164 (81.6)	
Bachelor degree	702 (58.5)	672 (95.7)		624 (88.9)	
Higher education	241 (20.1)	229 (95.0)		213 (88.4)	
Student					
Yes	457 (38.1)	441 (96.5)	0.001*	409 (89.5)	0.002*
No	743 (61.9)	679 (91.4)		618 (83.2)	
Student in healthcare					
Yes	263 (21.9)	257 (97.7)	0.001*	248 (94.3)	< 0.001*
No	194 (16.2)	184 (94.8)		161 (83.0)	
N/A	743 (61.9)	679 (91.4)		618 (83.2)	
Type of job					
Healthcare	171 (14.3)	167 (97.7)	< 0.001*	163 (95.3)	< 0.001*
Non-healthcare	475 (39.6)	423 (89.1)		383 (80.6)	
N/A	554 (46.2)	530 (95.7)		481 (86.8)	
Lost job due to COVID-19					
Yes	95 (7.9)	86 (90.5)	0.49	81 (85.3)	0.98
No	765 (63.8)	717 (93.7)		654 (85.5)	
N/A	340 (28.3)	317 (93.2)		292 (85.9)	
Province of residence					
Sindh	934 (77.8)	864 (92.5)	0.20	784 (83.9)	0.009*
Punjab	183 (15.3)	178 (97.3)		170 (92.9)	
Balochistan	27 (2.3)	26 (96.3)		26 (96.3)	
Khyber Pakhtunkhwa	43 (3.6)	40 (93.0)		35 (81.4)	
Gilgit and Baltistan	13 (1.1)	12 (92.3)		12 (92.3)	
Smoking status					
Smoker	149 (12.4)	135 (90.6)	0.04*	124 (83.2)	0.08
Never smoker	927 (77.3)	874 (94.3)		804 (86.7)	
Quit smoking	124 (10.3)	111 (89.5)		99 (79.8)	

*p < 0.05 is considered significant

Table 2 depicts the knowledge related to COVID-19. The majority (70.5%, *n* = 846) of the respondents answered correctly that COVID-19 is transmitted through air droplets and contact. A considerable (89.6%, *n* = 1075) part of the survey acknowledged that COVID-19 can lead to pneumonia and respiratory failure and more than three fourths (79.2%, *n* = 950) agreed that supportive care is the current treatment. Moreover, almost the entire population (97.8%, *n* = 1173) were aware of preventive measures. Overall, 93.3% (*n* = 1120) of our sample exhibited adequate knowledge of COVID-19 with a mean score of 6.59 ± 1.35 (range: 0–8).

**Table 2:** Knowledge and perceptions of COVID-19

	Yes	No	I do not know
KNOWLEDGE			
COVID-19 is thought to be originated from animals including bats and pangolins?	701 (58.4)	168 (14)	331 (27.6)
COVID-19 is transmitted through air droplets and contact?	846 (70.5)	233 (19.4)	121 (10.1)
Headache, fever, cough, sore throat, and flu are common symptoms of COVID-19?	1114 (92.8)	42 (3.5)	44 (3.7)
The period between exposure to the infection and appearance of symptoms (incubation period) of COVID-19 is 2-14 days?	1085 (90.4)	27 (2.3)	88 (7.3)
COVID-19 can lead to pneumonia, and respiratory failure?	1075 (89.6)	28 (2.3)	97 (8.1)
Supportive care is the current treatment for COVID-19?	950 (79.2)	70 (5.8)	180 (15)
Covering nose and mouth while coughing, and avoiding sick contacts and crowded places such as train stations and public transportations can help in the prevention of COVID-19 transmission?	1173 (97.8)	10 (0.8)	17 (1.4)
Ordinary residents can wear general medical masks to prevent the infection by the COVID-19 virus?	957 (79.8)	163 (13.6)	80 (6.7)
Knowledge score; mean ± SD; range	6.59 ± 1.35; 0–8		
Adequate knowledge	1120 (93.3)	80 (6.7)	
PERCEPTION			
Those who are elderly or chronically ill are more likely to be severely affected?	836 (69.7)	280 (23.3)	84 (7.0)
COVID-19 can be fatal?	1059 (88.3)	53 (4.4)	88 (7.3)
Washing hands with soap and water can help in the prevention of COVID-19 transmission?	1181 (98.4)	7 (0.6)	12 (1.0)
Flu vaccination is sufficient for preventing COVID-19?	51 (4.3)	905 (75.4)	244 (20.3)
Sick patients should share their recent travel history with healthcare providers?	1162 (96.8)	16 (1.3)	22 (1.8)
Perception score; mean ± SD; range	4.29 ± 0.82; 0–5		
Good perception	1027 (85.6)	173 (14.4)	

Table 3 shows the overall attitude and practices regarding COVID-19. Though, more than half (58.2%, *n* = 698) of the population was following the quarantine regulations imposed by the government ‘to a great extent’; fairly 36.8% (*n* = 441) showed just a ‘neutral’ attitude regarding the competency of the government of Pakistan in controlling the pandemic. Fear levels regarding infection of COVID-19 varied as 32.8% (*n* = 394) were afraid of themselves and their families from getting infected. An optimistic attitude was observed as 67.3% (*n* = 807) agreed that COVID-19 will be successfully controlled.

**Table 3:** Attitude and Practices towards COVID-19

	n (%)
How soon will the vaccine for COVID-19 develop?	
In a year or more	504 (42)
In the next few months	377 (31.4)
Now	21 (1.8)
Not possible to create a vaccine	16 (1.3)
Not sure	282 (23.5)
According to you, how much of the news and information about COVID-19 is made-up?	
A lot	435 (36.3)
Some	577 (48.1)
Not much	148 (12.3)
Not at all	40 (3.3)
Do you agree that ‘God has control over the spread of COVID-19’; therefore congregational prayers in the country cannot be a source of infection?	
Agree	245 (20.4)
Disagree	773 (64.4)
I don’t know	182 (15.2)
To what extent are you following the quarantine regulations imposed by the government?	
Not at all	27 (2.3)
To some extent	154 (12.8)
To a moderate extent	321 (26.8)
To a great extent	698 (58.2)
To what extent do you agree/disagree that the Government of Pakistan is controlling the COVID-19 situation very well?	
Strongly disagree	80 (6.7)
Disagree	170 (14.2)
Neutral	441 (36.8)
Agree	431 (35.9)
Strongly agree	78 (6.5)
In recent days, have you worn a mask when leaving home?	
Yes	1020 (85)
No	93 (7.8)
Never left home	87 (7.2)
To what extent do you agree or disagree with the following statement ‘I am afraid that I & someone in my household will be infected by COVID-19’?	
Strongly disagree	76 (6.3)
Disagree	138 (11.5)
Neutral	373 (31.1)
Agree	394 (32.8)
Strongly agree	219 (18.3)
Do you agree that COVID-19 will be successfully controlled?	
Agree	807 (67.3)
Disagree	78 (6.5)
I don’t know	315 (26.3)

Majority (85.6%, *n* = 1027) of the Pakistani residents showed good perception of COVID-19 with a mean score of 4.29 ± 0.82 (range: 0–5). As indicated by Table 2, almost all participants knew that washing hands with soap and water can help in the prevention of COVID-19 transmission (98.4%, *n* = 1181) and that sick patients should share their recent travel history with health care (96.8%, *n* = 1162). However, roughly one third (30.3%, *n* = 364) of the sample was not aware that elderly and chronically ill patients were more likely to be severely affected and nearly a quarter (24.6%, *n* = 295) of the respondents did not know that flu vaccination is not sufficient for preventing COVID-19.

Significant differences (*p* < 0.05) in knowledge and perception across both genders as well as among all age groups, education groups and between students and employees in the healthcare and non-healthcare department were noted (Table 1). Across province of residence, there was a significant difference in perception (*p* < 0.05). Likewise, a significant difference in knowledge (*p* < 0.05) between smokers and non-smokers existed.

On multivariate analysis for knowledge, age groups of 30–39 and 50–59 years had significantly lower knowledge. Female gender and an education level of minimum bachelors were significantly associated with adequate knowledge (Table 4). Likewise, female gender, a minimum education level of bachelor’s and being employed in the healthcare system was significantly associated with good perception (Table 5). The Cronbach’s alpha coefficient for the Knowledge scale was 0.771 and for the Perception scale was 0.697.

**Table 4:** Univariate and Multivariate logistic regression for knowledge

	**Odds ratio (95% CI)**	***p*** **value**
UNIVARIATE ANALYSIS		
Age		
15–19	1.0	
20–29	2.54 (1.14–5.65)	0.02*
30–39	0.80 (0.36–1.78)	0.59
40–49	1.24 (0.49–3.18)	0.64
50–59	0.70 (0.29–1.66)	0.42
60+	0.69 (0.27–1.73)	0.43
Gender		
Female	1.0	
Male	0.25 (0.15–0.41)	< 0.001*
Marital status		
Married	1.0	
Single	2.20 (1.38–3.50)	0.001*
Education		
No education	1.0	
Primary school	0.63 (0.20–1.99)	0.43
Secondary school	3.33 (1.24–8.90)	0.01*
Bachelor degree	8.25 (3.22–21.13)	< 0.001*
Higher education	7.03 (2.47–19.95)	< 0.001*
Student		
No	1.0	
Yes	2.59 (1.48–4.55)	0.001*
Type of job; *n* = 646		
Non-healthcare	1.0	
Healthcare	5.13 (1.82–14.41)	0.002*
Lost job due to COVID-19		
No	1.0	
Yes	0.64 (0.30–1.34)	0.24
Province of residence		
Sindh	1.0	
Punjab	2.88 (14–7.24)	0.02*
Balochistan	2.10 (0.28–15.75)	0.46
Khyber Pakhtunkhwa	1.08 (0.32–3.58)	0.90
Gilgit and Baltistan	0.97 (0.12–7.58)	0.97
Smoking status		
Smoker	1.0	
Never smoker	1.71 (0.92–3.16)	0.08
Quit smoking	0.88 (0.40–1.96)	0.76
	**Adjusted Odds ratio (95% CI)**	***p*** **value**
MULTIVARIATE ANALYSIS		
Age		
15–19	1.0	
20–29	1.06 (0.43–2.58)	0.89
30–39	0.37 (0.14–0.94)	0.03*
40–49	0.63 (0.22–1.78)	0.38
50–59	0.32 (0.12–0.87)	0.02*
60+	0.37 (0.13–1.04)	0.06
Gender		
Female	1.0	
Male	0.33 (0.20–0.57)	< 0.001*
Education		
No education	1.0	
Primary school	0.71 (0.21–2.35)	0.58
Secondary school	1.73 (0.60–4.97)	0.30
Bachelor degree	5.37 (2.01–14.36)	0.001*
Higher education	6.47 (2.21–18.98)	0.001*

*CI* Confidence Interval

*p < 0.05 is considered significant

**Table 5:** Univariate and Multivariate logistic regression for perception

	**Odds ratio (95% CI)**	***p*** **value**
UNIVARIATE ANALYSIS		
Age		
15–19	1.0	
20–29	1.80 (1.03–3.15)	0.03*
30–39	0.86 (0.47–1.57)	0.63
40–49	1.006 (0.51–1.95)	0.98
50–59	0.83 (0.42–1.61)	0.58
60+	0.66 (0.33–1.31)	0.23
Gender		
Male	1.0	
Female	2.09 (1.51–2.90)	< 0.001*
Marital status		
Married	1.0	
Single	1.84 (1.33–2.55)	< 0.001*
Education		
No education	1.0	
Primary school	1.02 (0.35–2.92)	0.96
Secondary school	5.17 (2.21–12.09)	< 0.001*
Bachelor degree	9.33 (4.16–20.90)	< 0.001*
Higher education	8.87 (3.73–21.09)	< 0.001*
Student		
No	1.0	
Yes	1.72 (1.20–2.45)	0.003*
Type of job *n* = 646		
Non-healthcare	1.0	
Healthcare	4.89 (2.32–10.31)	< 0.001*
Lost job due to COVID-		
19	1.0	
No	0.98 (0.53–1.79)	0.95
Yes		
Province of residence		
Sindh	1.0	
Punjab	2.50 (1.38–4.51)	0.002*
Balochistan	4.97 (0.67–36.93)	0.11
Khyber Pakhtunkhwa	0.83 (0.38–1.84)	0.65
Gilgit and Baltistan	2.29 (0.29–17.78)	0.42
Smoking status		
Smoker	1.0	
Never smoker	1.31 (0.82–2.10)	0.24
Quit smoking	0.79 (0.43–1.47)	0.47
	**Adjusted Odds ratio (95% CI)**	***p*** **value**
MULTIVARIATE ANALYSIS		
Gender		
Male	1.0	
Female	2.07 (1.25–3.44)	0.004*
Education		
No education	1.0	
Primary school	1.42 (0.46–4.41)	0.53
Secondary school	3.51 (1.06–11.52)	0.03*
Bachelor degree	5.53 (2.29–13.35)	< 0.001*
Higher education	7.74 (3.003–19.99)	< 0.001*
Type of job		
Non-healthcare	1.0	
Healthcare	2.90 (1.32–6.39)	0.008*

*CI* Confidence Interval

*p < 0.05 is considered significant

## Discussion

Epidemics and pandemics are occasional circumstances, and they bring multiple challenges for the affected population. Paucity of knowledge may lead to nonchalant behavior making it difficult to curb the disease. Pakistan, with its poor infrastructure, lack of emergency preparedness mechanisms and substandard testing rates struggles to combat the coronavirus. The number of recorded cases has grown exponentially, especially after the lockdown imposed in March 2020 was eased in May 2020 due to religious festivities; with a daily rise of approximately 1000 cases per million population. Punjab and southern Sindh provinces which make up 75% of the total cases in Pakistan, have slightly over only 14,000 beds for COVID-19 patients at state-run and private hospitals [10], causing most of the patients with milder symptoms to be managed at home instead. Moreover, the total number of functional ventilators in the country is just 1650 with varying numbers among provinces [11]. As a result, in an attempt to reform the health sector, Pakistan has inaugurated its first ever local production of ventilators, with an average manufacturing capacity of 250–300 units per month [12].

Research has proven good knowledge to be a significant predictor of correct practices in infection control [13, 14], while highlighting how a knowledge deficit can lead to an inadequacy [8]. The overall adequate knowledge of COVID-19 reported in our survey was 93.3%, which is higher than that in previous studies from Pakistan [15, 16]. This is parallel to a survey conducted in Tanzania where 84.4% had good knowledge [17]. Such figures did not come out as a surprise as the government of Pakistan has taken all the appropriate actions of updating their webpage, providing information regarding prevention and guidelines for the public. Informative television channels, numerous awareness campaigns by local nongovernmental organizations alongside the launch of healthcare related applications over mobile platforms have played a remarkable role in educating the citizens about the nature of the disease. Additionally, about 80% of the study population had a minimum education level of bachelor’s which may account for their high level of knowledge; this is further confirmed by the significant association of education level with adequate knowledge-similar to a study conducted in China [8]. It is interesting to note that the mean knowledge score for this sample was about 82% (6.59/8) which was quite comparable to the more developed parts of the world such as the United States [18] and China [8] with around 80 and 90% mean scores respectively.

In particular, the knowledge regarding symptoms of COVID-19 was good where about 93% were well-aware and around 79% knew that there is only supportive treatment available for the virus; both these findings were in accordance with a study from Jordan [19]. On the other hand, only 70.5% of the sample agreed that the virus spreads through air droplets and contact, whereas a similar study from Egypt [20] showed a wholesome 95% of the population to be aware of the same. About 14% of the sample believed that wearing medical masks does not protect against infection, which is noteworthy, as a report on health care workers reported about 17% to believe the same [21]. On the other hand, where almost 80% from this study agreed that wearing a mask offers protection, only 35% from a study in Egypt had parallel views [20]. These positive findings explain how the seriousness of the disease has been highlighted by multiple media and health platforms during the pandemic, successfully reaching the masses in the country. A study on health care workers from Uganda [21] reported a poor attitude towards COVID-19. However, a study among Malaysian [22], Chinese [8] and Vietnamese [23] citizens showed positive attitudes towards overcoming the COVID-19 crisis.

Despite much less faith in the government of Pakistan, 67.3% were optimistic that COVID-19 would be successfully controlled; though this was relatively low as compared to that in China [8] and Malaysia [22]. The government of Pakistan has taken several actions to limit the dispersion of the virus. Some important measures include suspension of all incoming flights at airports, closure of educational institutions and import of 1000 ventilators until the end of June 2020 [24]. However, despite these extensive preventative measures, 36.8% of the participants showed just a ‘neutral’ attitude regarding the competency of the government of Pakistan in controlling the pandemic. This wasn’t surprising as lockdown restrictions were soon eased and businesses were allowed to operate even though cases were on a rise, leading to the selling of medicines and personal protective items at higher than standard rates illegally [25]. Additionally, the aggressive media and the constant protests by political and religious leaders towards the government’s shortfall in controlling the pandemic played an important role in orchestrating the pessimism among citizens [26].

Nonetheless, Pakistan being a LMIC needed to take into account the consequences of an economic downfall due to a complete lockdown and so had little choice but to ease it, since most of the population survives on daily or monthly wages. However, as part of its continued anti-corona strategy, Pakistan’s implementation of a limited locality based lockdown (‘smart lockdown’) instead fairly proved to be successful as the number of cases and mortality rates related to COVID-19 relatively decreased in July 2020 [27]. Even so, the United Nations estimates that developing countries will need $2.5 trillion in rescue funding to avoid an economic and health catastrophe [28]. A significant burden on LMIC including Pakistan exists, as it struggles to overcome the constraints associated with improving healthcare reforms along with constantly striving to revive the weakening economy.

A study on Iranian medical students indicated that their women were more aware of the significance of the virus [29], in line with the multivariate analysis from this synthesis. Older participants (of age groups 50 to 59 years) had significantly lower knowledge scores, similar to a study from Egypt [20]. To the contrary, Malaysian citizens above the age of 50 years displayed higher knowledge [30] while another one in Vietnam among health care professionals did not show a relation between age and knowledge [23]. Discrepancies in knowledge across the demographics may have also prevented systemized attempts in decreasing the spread of the virus in Pakistan. For future purposes, it is important for policy makers to design strategies regarding provision of health care awareness resources and guidelines to help fix the gap in knowledge scores among the vulnerable demographics.

Lack of knowledge identified according to demographic characteristics can help us target specific populations to increase the overall awareness of the country. Focus should be on education of people above 30 years of age, females and the less literate population that is, those who have education of less than secondary school since they have significantly lower knowledge scores than others. Poor knowledge poses them a possibly higher risk of acquiring the virus so emphasis should be laid on maintaining precautions for this certain population to control the spread more effectively.

This survey also indicated a significant difference in knowledge between smokers and non-smokers. According to a recent survey conducted by Pakistan Alliance for Nicotine and Tobacco Harm Reduction, 66.7% of smokers were not willing to change their smoking habits during the pandemic and only 41.7% of them believed that smoking increased the risk of getting COVID-19 [31]. This emphasizes the need to raise further awareness in the Pakistani population regarding the severity of COVID-19 symptoms among smokers as many studies in the literature have revealed the same. In a report on COVID-19 infected individuals from China, 12.4% of current smokers and 23.8% of past smokers developed critical disease as compared to only 4.7% of those who had never smoked [32].

## Limitations

The study has some limitations. The sample size is acceptably large but might not be enough to be representative of the entire Pakistani population, so the findings should be generalized with caution. The participants were approached through convenience sampling through networks of the data collectors and circulated via multiple social media applications. Additionally, most of the participants are from the younger age group (20–39 years). This might be because the survey was mainly distributed online with 76 million internet users in Pakistan, out of which 63% belong to the ages between 20 and 25 years, according to a survey by Pakistan Telecommunication Authority [33]. The survey also mostly included the educated class with most of the respondents bearing a bachelor’s degree at minimum and majority (77.4%) of the population residing in an urban metropolitan city like Karachi; this might have led to an overestimation of knowledge and perception of Pakistan’s population. Furthermore, the authors were unable to collect equal responses from all provinces of the country as majority of the authors belonged to one province; therefore, one must be cautious in interpreting the results as generalizable for the whole country.

## Conclusions

Although the survey shows an overall good knowledge and perception of participants, certain demographics are identifiably less aware than the other with Pakistanis bearing diversified attitudes towards the pandemic. Moreover, considering the surge in COVID-19 cases in Pakistan, there lies a possibility of potential noncompliance in practices towards following preventive protocols. There is a need to follow these protocols and disseminate correct information through conducting elaborate awareness campaigns and educational interventions that target safe health practices and correct evidence-based information on this infection.

## Supplementary Information

**Additional file 1.**

## Data Availability

The datasets used and/or analyzed during the current study are available from the corresponding author on reasonable request.

## Abbreviations

COVID-19: Novel Coronavirus Disease
SARS-CoV-2: Severe acute respiratory syndrome coronavirus 2
WHO: World Health Organization
LMIC: Lower middle income countries
CI: Confidence interval
SPSS: Statistical Package for the Social Sciences
OR: Odds ratio
