# Supplementary material for: Knowledge, attitude and perception of Pakistanis towards COVID-19; a large cross-sectional survey
Source: BMC Public Health. 2021 Jan 5;21:21. doi: 10.1186/s12889-020-10083-y (PMC7783502; doi:10.1186/s12889-020-10083-y)
Supplement: Supplementary file 1 — Additional file 1. [file 12889_2020_10083_MOESM1_ESM.docx]

**Questionnaire**

Consent acknowledgement: We are conducting a cross-sectional study to assess the knowledge, attitude and perceptions (KAP) about the recent global COVID-19 pandemic among the general population of Pakistan. All information collected is exclusively used for the purpose of well-intentioned medical research and will not be shared beyond the scope of our research requirements. Please answer the following questions to the best of your knowledge. Once you complete the survey, you will be able to view the correct answers by clicking on “view accuracy”. Thank you for your participation.

1. Age:
2. 15-19 years
3. 20-29 years
4. 30-39 years
5. 40-49 years
6. 50-59 years
7. > 60 years
8. Gender: 1. Male. 2. Female 3. Do not wish to report
9. Marital status: 1. Married. 2. Single. 3. Divorced
10. Education: 1. No education. 2. Primary School. 3. Secondary School.

4. Bachelor’s degree. 5. Master’s degree

1. Are you a student? 1. Yes. 2. No
2. Are you a student in the health care field? 1. Yes. 2. No. 3. Not applicable
3. Type of job: 1. Healthcare 2. Non-healthcare 3. Not applicable
4. Did you lose your job/daily wage due to COVID-19? 1. Yes 2. No 3. Not applicable
5. City of living:
6. Have you smoked tobacco cigarette?

1) Yes, I am a smoker

2) Never smoked

3) I have quit smoking

1. Did you contract the novel COVID-19?
2. Yes
3. No
4. I don’t know
5. Has anyone of your close contact tested positive for COVID-19?
   1. Yes
   2. No
   3. I don’t know
6. COVID-19 is thought to be originated from animals including bats and pangolins?
   1. Yes
   2. No
   3. I don’t know
7. COVID-19 is transmitted through air droplets and contact?
8. Yes
9. No
10. I don’t know
11. Headache, fever, cough, sore throat, and flu are common symptoms of COVID-19?
    1. Yes
    2. No
    3. I don’t know
12. The period between exposure to the infection and appearance of symptoms (incubation period) of COVID-19 is 2-14days?
    1. Yes
    2. No
    3. I don’t know
13. COVID-19 can lead to pneumonia, and respiratory failure?
    1. Yes
    2. No
    3. I don’t know
14. Those who are elderly or chronically ill are more likely to be severely affected?
    1. Yes
    2. No
    3. I don’t know
15. Supportive care is the current treatment for COVID-19?
    1. Yes
    2. No
    3. I don’t know
16. COVID-19 can be fatal?
    1. Yes
    2. No
    3. I don’t know
17. Covering nose and mouth while coughing, and avoiding sick contacts and crowded places such as train stations and public transportations can help in the prevention of COVID-19 transmission?
    1. Yes
    2. No
    3. I don’t know
18. Washing hands with soap and water can help in the prevention of COVID-19 transmission?
    1. Yes
    2. No
    3. I don’t know
19. Ordinary residents can wear general medical masks to prevent the infection by the COVID-19 virus?
    1. Yes
    2. No
    3. I don’t know
20. Flu vaccination is sufficient for preventing COVID-19?
    1. Yes
    2. No
    3. I don’t know
21. Sick patients should share their recent travel history with healthcare providers?
    1. Yes
    2. No
    3. I don’t know
22. How soon will the vaccine for COVID-19 develop?
    1. In a year or more
    2. In the next few months
    3. Now
    4. Not possible to create a vaccine
    5. Not sure
23. According to you, how much of the news and information about COVID-19 is made-up?
    1. A lot
    2. Some
    3. Not much
    4. Not at all
24. Do you agree that ‘God has control over the spread of COVID-19’; therefore congregational prayers in the country cannot be a source of infection?
25. Agree
26. Disagree
27. I don’t know
28. To what extent are you following the quarantine regulations imposed by the government?
29. Not at all
30. To some extent
31. To a moderate extent
32. To a great extent
33. To what extent do you agree/disagree that the Government of Pakistan is controlling the COVID-19 situation very well?
34. Strongly disagree
35. Disagree
36. Neutral
37. Agree
38. Strongly agree
39. In recent days, have you worn a mask when leaving home?
    1. Yes
    2. No
    3. Never left home
40. To what extent do you agree or disagree with the following statement ‘I am afraid that I & someone in my household will be infected by COVID-19’?
41. Strongly disagree
42. Disagree
43. Neutral
44. Agree
45. Strongly agree
46. Do you agree that COVID-19 will be successfully controlled?
    1. Agree
    2. Disagree
    3. I don’t know
